# Comprehensive Study of Antibiotics and Antibiotic Resistance Genes in Wastewater and Impacted Mediterranean Water Environments

**DOI:** 10.3390/antibiotics14040341

**Published:** 2025-03-26

**Authors:** Maria Garcia-Torné, Irene Falcó, Xavier Borrell, Arianna Bautista, Rachida Mazigh, Rosa Aznar, Gloria Sánchez, Marinella Farré, Marta Llorca

**Affiliations:** 1 Institute of Environmental Assessment and Water Research, C/Jordi Girona, 18-26, 08034 Barcelona, Spain; mgtqam@cid.csic.es (M.G.-T.); xbdqam@cid.csic.es (X.B.); abgqsh@cid.csic.es (A.B.); rmlqsh@cid.csic.es (R.M.); mfuqam@cid.csic.es (M.F.); 2 Doctoral Program in Analytical Chemistry and Environmental Science, Department of Chemical Engineering and Analytical Chemistry, University of Barcelona, 08028 Barcelona, Spain; 3 VISAFELab Laboratory, Department of Preservation and Food Safety Technologies, IATA-CSIC, 46980 Valencia, Spain; irene.falco@iata.csic.es (I.F.); gloriasanchez@iata.csic.es (G.S.); 4 Department of Microbiology and Ecology, University of Valencia, 46100 Valencia, Spain; rosa.aznar@uv.es

**Keywords:** antibiotics, suspect screening, antibiotic resistance genes, antibiotic resistant bacteria, bacteria

## Abstract

**Background:** The spread of antimicrobial resistance is a central public health problem. Wastewater treatment plants and impacted environments are well-known hotspots for antibiotic resistance. However, there is still limited knowledge regarding where antibiotic resistance genes (ARGs) acquire mobility. **Method:** In this study, we aimed to gather evidence on the seasonal patterns of ARG spread in two Mediterranean areas from NE and E of Spain (Ebro River and Ebro Delta, and Xúquer River and Albufera de València), correlating ARG presence, with special focus on the faecal bacteria *Escherichia coli*, with antibiotic residues and environmental conditions. The analytical methodology employed was based on a suspect screening approach, while a novel prioritisation approach for antibiotics was proposed to identify those areas more susceptible to the spread of ARG. **Results:** Our findings demonstrate that ARG levels in wastewater were similar across different seasons, although a greater diversity of ARGs was recorded in summer. We hypothesise that horizontal gene transfer among aquatic bacterial populations during the northeastern Mediterranean summer, when temperatures reach approximately 35~40 °C, could be a key driver of ARG dissemination. By contrast, the highest concentrations of antibiotics in winter samples, with temperatures around 5~10 °C, may promote the spread of microbial resistance. **Conclusions:** Our key findings highlight that water temperature and sunlight irradiation are crucial factors influencing antibiotic levels and microbial abundance, requiring further investigation in future studies.

## 1. Introduction

Antibiotics have drastically changed modern medicine, extending the average human lifespan worldwide by 23 years [1]. However, the overuse and misuse of antibiotics and antimicrobials have rapidly increased antimicrobial resistance (AMR), to the point that some infections are now untreatable [2]. Thus, AMR has been recognised as a major global health challenge involving antibiotic residues and AMR transfer between humans, animals, and the environment [3].

The main routes of antibiotics in the environment are the discharge of antibiotic residues during production processes and after their usage in human medicine (i.e., through clinical and urban wastewater), as well as in veterinarian practices, including factory farming for food production and aquaculture. Wastewater treatment plants (WWTPs) are considered to be critical hotspots for the acquisition and environmental spread of AMR in the environment [4] because of the ubiquitous occurrence of antibiotic residues in wastewater, because antibiotic resistant bacteria (ARB) and antibiotic resistance genes (ARGs) are also present in wastewater, and because of the favourable conditions that promote the transfer of ARGs among bacteria [5,6]. In the environment, antibiotics can pose a selective pressure on bacteria, even at sub-inhibitory concentrations [7], leading to intrinsic AMR [8,9]. Therefore, the environment has a relevant role in the transmission and evolution of AMR [3,10,11]. In some instances, ARGs originate in the environmental microbiota [12]. Then, the analyses of different environmental matrices may provide valuable information about the acquired resistance in an area, especially when the samples are close to the emission sources [13]. Monitoring of both WWTPs’ antibiotic and AMR residues is particularly promising for surveillance, as WWTPs’ discharge contains pooled faecal bacteria from large populations, providing broad coverage, and also provides an early warning for the early spread of resistance factors [14]. However, other environmental biotic and abiotic factors can influence the development and spread of AMR [15].

During the last decade, many studies have reported the occurrence of antibiotics [16,17,18] and ARGs [19,20] in WWTPs, and there have been some integrative studies combining both aspects [5,21,22,23,24]. Some studies have applied more comprehensive approaches based on suspect screening [25,26,27], but in these cases, only antibiotics were assessed. So, more holistic studies are needed to extensively identify the whole set of drivers that rule the environmental evolution and transmission of antibiotic resistance, including rising temperatures, eutrophication processes, storms, and other contaminants in the same compartments. Moreover, AMR and the current situation of climate change are two interlinked global health priorities [28]. The increases in temperature and humidity are directly linked to AMR, as higher temperatures are associated with increased bacterial growth rates [29], and enhanced horizontal gene transfer [28,30]. For instance, *Salmonella* spp., which is becoming increasingly antibiotic resistant, is affected by these factors [31]. Moreover, seasonality plays a role in certain pathogens, as seen in the increased incidence of Gram-negative infections during the warmer months [32], reflecting optimal growth conditions for many Gram-negative bacteria at 32–36 °C. Despite these observations, there is a lack of studies examining the impact of seasonality on AMR. In this context, the Mediterranean region is particularly affected, as its temperature is warming 20% faster than the global average [33]. Regarding AMR, it has been proven that Mediterranean regions present an increase in resistance from north to South and from West to East, e.g., the particular case of resistance carried by some Gram-negative bacteria like *Klebsiella pneumoniae* and *Escherichia coli* to third-generation cephalosporin, often combined in opposition to fluoroquinolones and aminoglycosides [34]. On the other hand, the use of antibiotics in Mediterranean aquaculture has been proven to be a key factor for inducing antibiotic resistance in the surrounding bacteria in the water column, sediment, and fish-associated bacterial strains [35]. Then, ARGs are diffused through horizontal gene transfer.

In this context, the primary objective of the present study was to investigate the relationship between antibiotic residues and AMR in two impacted areas of the Mediterranean coast with high anthropogenic and climate pressures: the last part of the Ebro River from Zaragoza city to Ebro Delta National Park (NE of Spain), with a wetland area of around 7736 hectares; and Albufera National Park of Valencia (east of Spain), with freshwater lagoon area of around 2800 hectares. Here, we present a comprehensive assessment of climatic and anthropogenic pressures: we characterise the AMR (ARGs and ARB), simultaneously focusing on the faecal bacteria *E. coli*, and the seasonal profile of antibiotics, both in WWTPs and in impacted surface water. A suspect screening analysis, based on liquid chromatography coupled with high-resolution mass spectrometry (LC-HRMS), followed by confirmatory quantitative analysis of prioritised antibiotics, has been performed with a new approach that is designed to select antibiotics with a major impact, from the point of view of resistance.

## 2. Results and Discussion

### 2.1. Levels of ARGs

**Figure 1:**
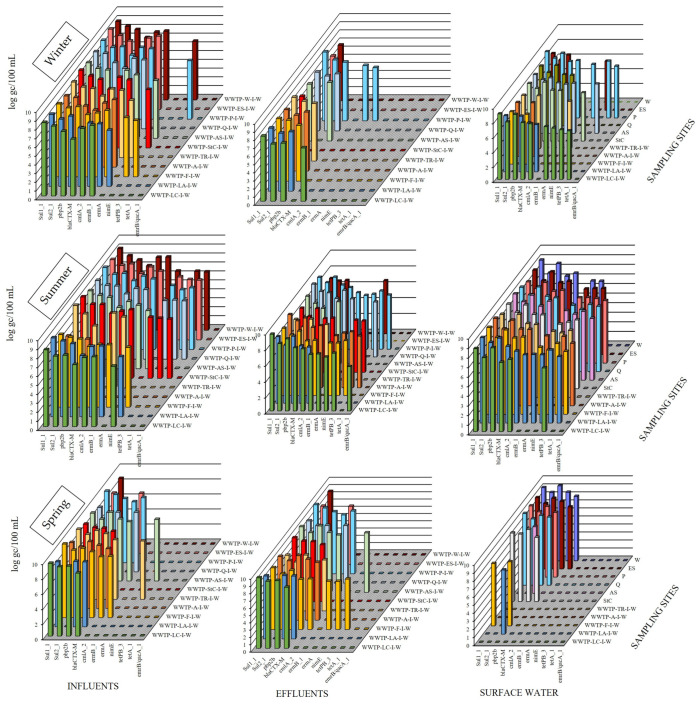
Levels of different antibiotic resistance genes (ARGs) in influent, effluent, and surface water samples. gc: genome copies, WWTPs: wastewater treatment plants.

Eleven resistance genes, which were selected among those resistance genes that are more commonly found in wastewater [36,37,38] in the Mediterranean area, were investigated by qPCR, according to the procedure that is described in the methods section of this paper [39,40]. The selected resistance genes were sulfonamide resistance genes (*sul1_1* and *sul2_1*), penicillin-binding protein (*pbp2b*), β-lactamases (*bla_CTX-M_*), acquired resistance to chloramphenicol gene (*cmlA_2*), macrolide—lincosamide—streptogramin B resistance genes (*ermB_1*, *ermA*, and *ermB/qacA*), nitroimidazole resistance gene (*nimE*), and acquired tetracycline resistance genes (*tetPB_3* and *tetA_1*). The 16S rRNA gene was used as a positive control for qPCR analysis and to normalise the abundances of the identified genes in water samples. In Figure 1, the distribution of the investigated ARGs is shown.

As observed, the highest levels and diversity of ARGs were detected in summer, with 100% of the target genes being detected, reaching 10.02 log gc/100 mL for *Sul1_1*. These findings correlated with higher temperatures and increased solar irradiation (see Table S5), followed by spring and winter. In the summer and spring samples, higher levels of ARG were observed in WWTP influents and in those effluents without tertiary treatment, while in winter, the WWTPs with higher levels were the ones supplying a higher number of inhabitants. The studied seasons presented significant differences, as can be seen in Table S9, in which the *p*-values were inferior to 0.05 (α = 0.05 or 5% of significance); moreover, *sul1_1, sul2_1, pbp2b*, and blaCTX-M had very significant *p*-values < 0.01 (α = 0.01 or 1%). Finally, the differences were highly significant in one case, *cmlA_2*, with *p* < 0.001. The most frequently found ARGs in the wastewater influents were *sul1_1, sul2_1*, *pbp2b, blaCTX-M*, *cmlA_2*, and *ermB*, with frequencies exceeding 85%, while the most frequently found ARGs in the effluents were *sul1_1*, *sul2_1*, and *pbp2b*. The highest dominance of *sul1_1* in wastewater was previously reported [41,42]. It should be mentioned that in certain WWTPs, highly similar levels of ARGs were found in the influents and effluents, or even slightly higher in the influents, as in the case of *pbp2b* and *bla*_CTX-M_ in the spring, without a noticeable decrease in levels between treated and untreated water. This change in summer was substantially more obvious in all of the ARGs tested, except *cmlA*_2.

The increased effluent concentrations may be attributed to the spread of ARGs among bacterial communities in activated sludge, facilitated by horizontal gene transfer (HGT) [37], especially at higher temperatures, which are conducive to microbial growth. Similarly, some ARGs, such as *bla*_CTX-M_, *ermB*_1, *ermA*, *nimE*, *tetPB*_3, or *tetA*_1, showed increased concentrations when comparing effluents with downstream river samples in winter. This also agreed with the work of Liu, X. et al. [20], in which the authors reported higher concentrations of ARGs in biofilm samples collected downstream of WWTP effluents, or with the work of Reichert, G. et al. [43], wherein it was reported that there were higher concentrations of ARGs downstream of WWTP effluents.

**Figure 2:**
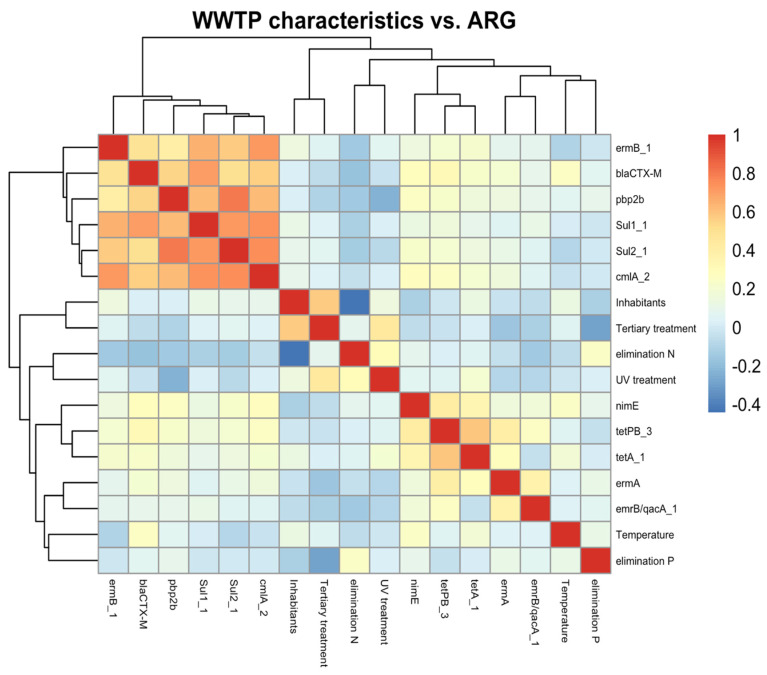
Correlation between wastewater effluents, WWTP characteristics, and ARG levels. Correlation performed in R software using R studio with pheatmap package version 1.0.12.

In Figure 2, a heatmap is presented that summarises the relationship between ARGs in the effluents and WWTP characteristics. In this graph, only effluents were included since treatment influenced the characteristics of the effluents. Cluster analysis was performed according to the Euclidean distance and Ward agglomeration approaches. As can be seen, considering only the effluents, the number of inhabitants treated in the different WWTPs almost did not influence the ARG levels. Tertiary treatment and P and N elimination processes showed inverse tendencies with ARG levels; however, according to the *p*-values e.g., for *bla*_CTX-M_, −0.247 cannot be considered significant with a signification value α = 0.05.

On the other hand, the ambient temperature correlated positively with some ARGs, such as *bla*_CTX-M_, *tetA_1*, and *nimE*. In general, in southern Europe, the percentage of antimicrobial resistance is higher than in northern countries. This can be attributed to higher population density and the influence of livestock farming. However, the irradiation and higher temperatures can also influence ARG spread, as observed here in the same areas and by comparing the levels throughout the year.

### 2.2. Quantification of E. coli and Extended Spectrum Beta-Lactamase-Producing E. coli (ESBL-E. coli)

**Figure 3:**
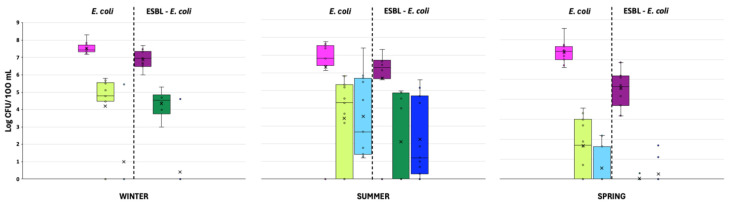
Average levels of the total *E. coli* (left light colours) and extended spectrum beta-lactamase-producing-*E. coli* (right dark colours) in influent (pink), effluent (green), and surface water (blue) samples grouped by season. CFU: colony forming unit.

The most substantial inter-sample variations in the effluent and surface waters were observed during summer and spring sampling (Figure 3 and Table S1).

**Figure 4:**
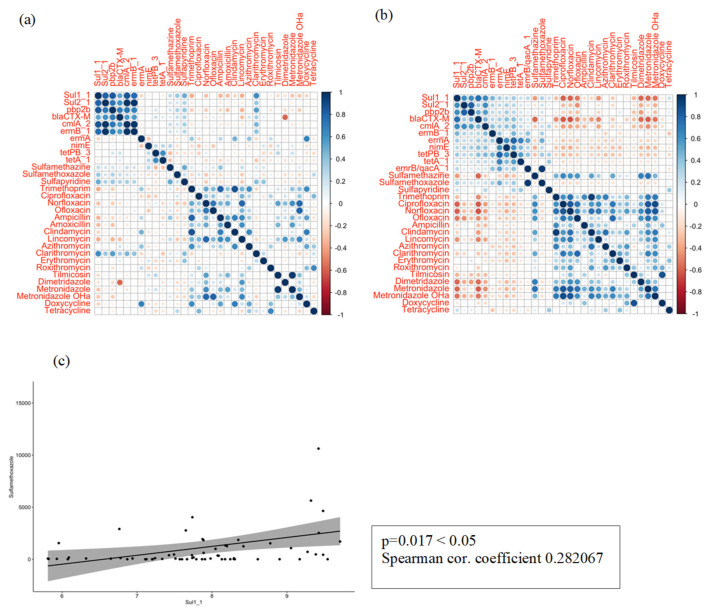
Correlations between ARGs and (**a**) influent (**b**) effluents and (**c**) example of linear correlation between ARGs and antibiotics.

The spatiotemporal distribution of *E. coli* and ESBL-*E. coli* counts are displayed in Figure 4. Both *E. coli* and ESBL-*E. coli* levels in influent and effluent waters showed similar concentrations across different seasons, except for spring, when the levels of *E. coli* in the effluents were lower and ESBL-*E. coli* was not detected. As for surface water, both bacteria were detected only in summer and sporadically in spring, when *E. coli* was present in some samples.

The reductions observed in the levels of both microorganisms between the influents and effluents followed the same pattern, with log reductions of approximately 2, 1, and 4 log CFU/100 mL during the winter, summer, and spring seasons, respectively. The averages levels of *E. coli* and ESBL-*E. coli* were 6.74 and 5.70, 3.97 and 3.08, and 3.68 and 2.83 log CFU/100 mL for the influent, effluent, and surface waters, respectively. Therefore, the seasons had a significant influence on these ARGs. These data aligned with data previously reported elsewhere [44], with 7.89 and 5.36 log CFU/100 mL for *E. coli* and ESBL-*E. coli* in influent samples, respectively, and 3.56 and 2.00 log CFU/100 mL in effluents for *E. coli* and ESBL-*E. coli*, respectively. While the reductions demonstrated by Haberecht H.B. et al. [44] did exceed the reductions observed in the present study (4.33 and 3.36 log for *E. coli* and ESBL-*E. coli*, respectively), they still fell short of the minimum reduction of 5 log that was established by the European Union as a validation requirement for the use of reclaimed water (EU 2020/741, 2020) [45]. These discrepancies may be attributed to the fact that the influent water sampling conducted by Haberecht H.B. et al. [44] was carried out near sewer areas where the accumulation of organic matter, and consequently microorganisms, was generally higher. Recent studies [41] found similar levels of ESBL-*E. coli* in influent samples, with concentrations of 5.00 and 5.88 log CFU/100 mL, respectively. On the contrary, levels of ESBL-producing *E. coli* in effluent waters were lower, with levels of 1.0 log CFU/100 mL or below the limit of detection (<LOD) [41], indicating better performance of the water reclamation treatment. Regarding the reclamation treatment implemented by the WWTPs, the overall mean reductions for *E. coli* and ESBL-*E. coli* were only 1.58 and 1.04 log, respectively. Again, these reductions do not meet the 5-log threshold that was recently established by the European Union (EU 2020/741, 2020) [45]. Consequently, there is a pressing need to enhance water reclamation processes. Furthermore, the observed reductions, which deviated substantially from the legally mandated thresholds (EU 2020/741, 2020) [45], underscore the low efficacy of WWTPs in mitigating the microbial load and addressing the potential risks associated with these pathogens (for example, pathogenicity and transmission of antibiotic resistance). This is a key factor that has been investigated during the last years. Although it is known that abiotic factors (e.g., temperature, pH, and conductivity, among others), play a crucial role in the bacterial community dynamics and, hence, on the propagation of ARB during wastewater treatment, it is not clear how these bio-physico-chemical factors or conditions could be used as predictors to estimate the efficiency of ARB removal that takes place during secondary treatments in WWTPs [4]. All of these findings emphasise the need to implement an effective wastewater treatment system, for instance, using UV light either alone or in conjunction with advanced oxidizing agents, such as chlorine and peracetic acid (PAA), to diminish the presence of *E. coli*-ESBL and thereby mitigate the dissemination of ARB through the food chain when used for irrigation [41].

### 2.3. Broad Assessment and Confirmation of Antibiotic Residues in Wastewater and Surface Water

A suspect screening analysis approach was applied to assess the main profile of antibiotic residues, followed by a targeted analysis of prioritised antibiotic residues. Suspect screening approaches are applied to environmental samples to search and tentatively identify chemical compounds by comparing the results against a spectral library or database, giving a wide comprehensive characterisation of selected environmental samples.

The antimicrobials found in the suspect screening belonged to different groups, i.e., sulphonamides, metronidazoles, macrolides, quinolones, and β-lactams, among others. Twenty-nine (29) compounds were tentatively identified in the winter wastewater influents and 25 compounds were tentatively identified in the effluents. Moreover, the compounds that were identified in different season presented the highest signal intensities in winter, indicating higher concentrations. This fact agreed with both the higher consumption of these pharmaceuticals in order to combat seasonal infections [46,47] and with the lower degradation rates due to lower sun irradiation and temperature during the treatment, as has been reported in previous works [46,47]. In summer, the lowest levels of antibiotic residues, tentatively identified at level 2, were found (24 in the influents and 16 in the effluents). This agreed with lower prescription use and higher sun irradiation per day, which promotes antibiotic transformations or degradation [48].

In spring, 34 antibiotics were tentatively identified in the influents and 25 antibiotics were tentatively identified in the effluents. Spring rains and the consequential farm runoff and soil resuspension can influence these levels. The tentatively detected compounds in each group of samples by season are summarised in Complementary Table S1.

The most dominant compounds found in the influents, which were present in more than 50% of the samples in all seasons, were antifolates, such as sulfamethoxazole and trimethoprim; fluoroquinolones, such as ofloxacin; macrolides, including azithromycin, clarithromycin, and tilmicosin; tetracyclines; and metronidazole. In the effluents, the compounds identified in more than 50% of the samples in all seasons were ofloxacin, azithromycin, and clarithromycin. Fewer compounds were detected in surface water, particularly in summer, with only 7 compounds identified at confidence level 2 in the Ebro River and 8 compounds identified at confidence level 2 in Albufera National Park. In springtime and winter, 9 and 19 antibiotic residues were detected in the Ebro River and 21 and 18 were detected in Albufera National Park, respectively. In contrast to wastewater, springtime showed a lower number of residues because, despite the number of antibiotics being higher in wastewater, the concentrations were lower than in other seasons, and then, once diluted in the environment, the concentrations of the residues were below the limit of detection.

In coastal water, seven antibiotics and 2 antiviral agents were detected. Among them ormetoprim and ronidazole were not detected in wastewater or surface water, even though they are antibiotics that are mainly used in veterinary medicine, including aquaculture. Moreover, azithromycin, ofloxacin, and tetracycline were present in more than 50% of the studied seawater samples. A summary of the different antibiotics and their frequencies are reported in Supplementary Material Table S2.

### 2.4. Prioritisation of Antimicrobial Residues

The antibiotic residues that were tentatively identified at level 2 were prioritised using a new approach that was designed to select those antibiotic residues with higher relevance in inducing AMR. This approach considers the frequency of antimicrobial detection in WWTP influents and effluents. The minimum predicted no-effect concentration (PNEC), according to the data provided by NORMAN-Network [49], refers to the percentage of resistance isolates per antibiotic class in 8 selected pathogens, including *Acinetobacter baumannii*, *Enterococcus faecalis*, *Enterococcus faecium*, *E. coli*, *Klebsiella pneumoniae*, *Pseudomonas aeruginosa*, *Streptococcus pneumoniae*, and *Salmonella* spp., which were selected according to their impact in terms of burden, transmissibility, treatability, and prevention options. The percentage of resistance isolates per antibiotic/pathogens pair was obtained from the European Antibiotic Resistant Surveillance Network (EARS-Net) (https://www.ecdc.europa.eu/en/sitemap, accessed on 12 December 2024), the World Health Organization (WHO) Bacterial Priority Pathogens List, 2024 (https://www.who.int/health-topics/antimicrobial-resistance, accessed on 12 December 2024), the ATLAS database (www.atlas-surveillance.com, accessed on 12 December 2024), and other recent publications [50,51]. Finally, the impact of antibiotics in animal production was also assessed in accordance with the European Medicines Agency recommendation. Table 1 summarises the score ranges employed, which range from 0 to 4 for each parameter, with 0 being the lowest impact and 4 being the highest. Supplementary Material Table S3 summarises the prioritisation scores for each antimicrobial that was tentatively identified.

Compounds with a score greater than 7 were prioritised, confirmed, and quantified by standards. Tetracycline, amoxicillin, sulfamethoxazole, roxithromycin, and ciprofloxacin showed the highest values, in agreement with the classification of antibiotics for evaluation and monitoring of use (2021) of the WHO Access Watch Reserve (AWaRe) [52] in which tetracycline is set as the most impacting compound, while roxithromycin is classified as a watch group antibiotic.

### 2.5. Confirmation and Quantification of Antimicrobial Residues

The target analysis of prioritised compounds was carried out by LC-HRMS in data-dependent acquisition (DDA) mode. The main analytical parameters are presented in Supplementary Material Table S4. In Table 2, a summary of quantified antimicrobials is presented. The detailed results of the different samples are presented in Complementary Table S2.

The compounds that were detected and confirmed in wastewater influents were sulfamethoxazole, ciprofloxacin, ofloxacin, and tetracycline, and the compounds that were detected and confirmed in the effluents were sulfapyridine, ofloxacin, ciprofloxacin, and tetracycline. Regarding the maximum levels, they were reported for tilmicosin (79 μg/L) in winter and ciprofloxacin (22 μg/L) in spring when, despite a generally wide variety of antimicrobials, they were detected but at lower levels.

### 2.6. Correlations Between Antibiotics and ARGs

We conducted a correlation analysis between the absolute concentrations of ARGs and the antibiotics in wastewater to which they conferred resistance, in order to determine potential links between both variables. As seen in Figure 4a, no correlations were evident when using the Pearson correlation coefficient (PCC) approach and considering all sampling campaigns. The levels of ARGs were similar throughout the year. This can be explained by the fact that once resistance is acquired, it can be transferred seasonally and regardless of the antibiotics’ presence [53]. Moreover, WWTPs receive sewage from various sources, promoting the interaction of bacteria and the exchange of genes horizontally, particularly in summer, when bacterial growth is favoured. Therefore, in summer, when antibiotic degradation is promoted and concentrations are lower, horizontal transfer is the favoured mechanism. However, in winter, when the concentration of antibiotics is higher and degradation is slower, correlations between antibiotic concentrations and ARGs were found, as seen in Figure 4b. For example, in the case of *sul1_1*, it presented a weakly positive correlation with sulfamethoxazole (*p* = 0.20). On the other hand, *blaCTX-M* presented a significantly negative correlation with norfloxacin (*p* = 0.45). Then, the monotonic relation between ARGs and antibiotic residues was explored using Spearman correlation analysis. In this case, Spearman correlation analysis was applied because the relationship between the variables was monotonic (i.e., consistently increasing or decreasing) but not necessarily linear. As can be seen in the selected examples of Figure 4c, a moderate correlation was found between sulfamethoxazole and *sul1_1*, and a strongly positive correlation was found between lincomycin and *erm_1*. These results were consistent with previous studies, suggesting that antibiotic exposure leads to selective pressure for ARGs [37,54,55].

**Figure 5:**
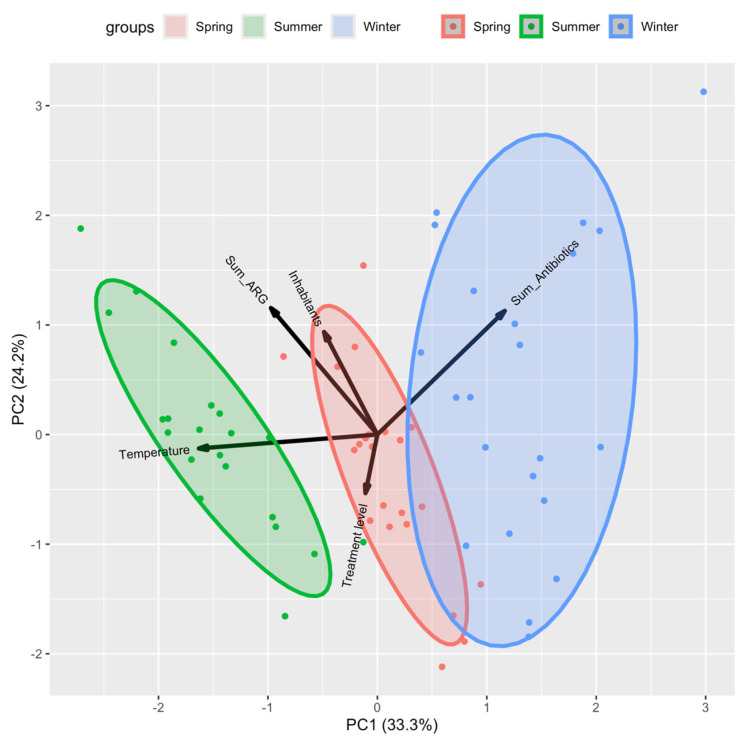
Principal component analysis of general WWTP characteristics, physicochemical characteristics, sum of antibiotics quantified, and sum of ARG copies.

In general terms, as can be seen in Figure 5, the temperature and the treatment levels were negatively correlated with the sum of antibiotics, while the ARG levels were higher in WWTPs that supplied a higher number of inhabitants.

## 3. Materials and Methods

### 3.1. Sampling Strategy

**Figure 6:**
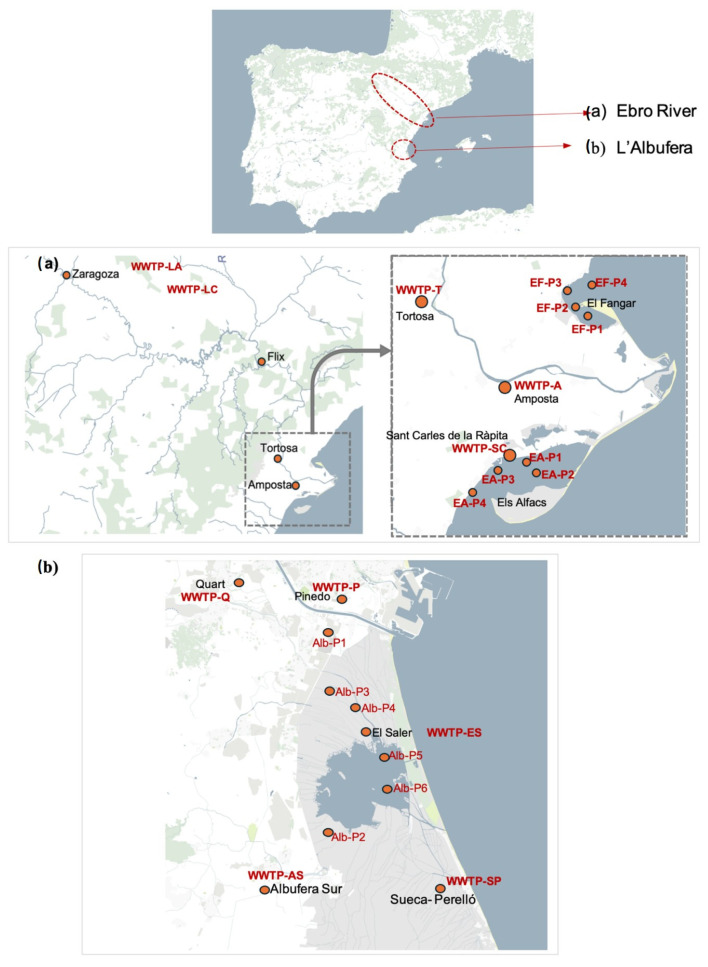
Sampling locations (**a**) the Ebro River and (**b**) Albufera National Park.

Among its many environmental impacts, climate change creates favourable conditions for the spread of ARM, and the Mediterranean region has been identified as a particularly vulnerable region in this sense [35,56]. The present study assessed two particularly impacted areas that are located in the NE and East of Spain under different seasonal conditions in order to evaluate the relationships between antibiotic occurrence, ARGs, ARB, and other climatic and socioeconomic conditions of the Mediterranean region. The first area comprised the end of the Ebro River, from Zaragoza city to the mouth of the Ebro River, including its coastal water. The second area comprised the aforementioned Albufera National Park in Valencia, on the Gulf of Valencia coast. Both areas are impacted by anthropogenic pressures such as WWTP effluents, farming, agriculture, and tourism. The two areas are shown in Figure 6.

The different sampling campaigns were performed in January 2022, July 2022, and March 2023, as the winter, summer, and spring campaigns, respectively. Despite all locations sharing typical Mediterranean characteristics, slight differences can be observed regarding local temperatures or precipitation regimes. For further information, see Table S5 [57,58,59,60,61,62].

A total of 106 water samples were analysed, comprising the influents and effluents of eleven WWTPs, surface water collected about 1 km away from WWTP discharge, marine water of the two marine bays of the Ebro Delta, together with aquaculture impact. Detailed information on the sampling sites and collection is provided in the Supplementary Material Table S6.

### 3.2. Water Sample Concentration for ARG Analysis and DNA Extraction

To concentrate the influent and effluent wastewater samples (200 mL), an aluminum-based adsorption precipitation method was performed [39]. Surface water samples (5 L) were concentrated by a Dead-End Hollow Fibre Ultrafiltration (DEUF) procedure as previously described elsewhere [40], using Rexeed-25A dialysis filters (Asahi Kasei Medical Co., Ltd., Tokyo, Japan). Concentrated samples were kept frozen at −80 °C.

Nucleic acid extraction from the concentrated samples was performed using the Maxwell^®^ RSC Instrument (Promega, Madrid, Spain) with the Maxwell RSC Pure Food GMO and authentication kit (Promega, Madrid, Spain) and the “Maxwell RSC Viral Total Nucleic Acid” running programme. Ultimately, DNA was eluted in 100 μL of nuclease-free water. The extraction procedure included a negative control consisting of nuclease-free water instead of concentrated sample.

### 3.3. High-Throughput qPCR Analysis

A total of eleven resistance genes were investigated in this study, which were selected among those resistance genes that are most commonly found in wastewater in the Mediterranean area, including acquired sulfonamide resistance genes (*sul1_1* and *sul2_1*), penicillin-binding protein (*pbp2b*), β-lactamase (*bla_CTX-M_*), acquired resistance to chloramphenicol gene (*cmlA_2*), macrolide–lincosamide–streptogramin B resistance genes (*ermB_1, ermA*, and *ermB/qacA*), nitroimidazole resistance gene (*nimE*), and acquired tetracycline resistance genes (*tetPB_3* and *tetA_1*). As a positive control for qPCR analysis and to normalise the abundances of the identified genes in the water samples, the 16S rRNA gene was used. Quantification of the selected genes was performed by high-throughput quantitative PCR (HT-qPCR) using the SmartChip^™^ Real-Time PCR system (TakaraBio, CA, USA) by Resistomap Oy (Helsinki, Finland). qPCR cycling conditions and processing of raw data are described elsewhere [63,64,65]. Each DNA sample was analysed in duplicate. Data processing and analysis were performed by using a Python-based script by Resistomap Oy (Helsinki, Finland) [66,67].

### 3.4. Quantification of E. coli and Extended Spectrum Beta-Lactamase-Producing E. coli

For influent and effluent wastewater samples, *E. coli* and extended spectrum beta-lactamase-producing *E. coli* (ESBL-*E. coli*) enumeration was assessed using selective culture media Chromocult coliform agar (Merck, Darmstadt, Germany) and CHROMagar ESBL (CHROMagar, Paris, France), respectively. Influent wastewater samples were diluted serially, and 0.1 mL aliquots were spread-plated. Effluent and surface water samples (200 mL) were filtered via a filtering ramp using 0.45 μm cellulose nitrate membrane filters (Sartorius, Madrid, Spain). Plates were then incubated at 37 °C for 24 h. Dark blue-violet colonies were considered positive for *E. coli*, while dark pink-reddish colonies were considered positive for ESBL-*E. coli*. The bacterial analyses were performed in duplicate, and the results were expressed as colony forming units (CFU)/100 mL. The detection limit (LOD) for *E. coli* and ESBL-*E. coli* counts in the influent samples was 1.0 Log CFU/100 mL (100 CFU/100 mL), while in the effluents, the LOD was 0 Log CFU/100 mL (1 CFU/100 mL).

### 3.5. Sample Pre-Treatment and Extraction for the Analysis of Antibiotics

The antibiotic standards for target analysis purposes are summarised in Supplementary Material Table S7.

Sample pre-treatment was based on a methodology that is described elsewhere [68], with minor modifications. Briefly, samples were spiked with a labelled internal standard mixed solution and filtered through a 0.7 μm filter. EDTA (0.1 M) was added to a final concentration of 0.1% (g solute/g solution) to minimise the chelation of macrolides, and the sample was then acidified with concentrated HCl to pH 2.5. Then, 25, 50, 100, and 200 mL of WWTP influents, WWTP effluents, surface water, and seawater were extracted, respectively. Oasis HLB (60 mg, 3 mL) cartridges were used for the SPE of WWTP, river, and Albufera samples, while Oasis HLB (200 mg, 6 mL) cartridges were used for sea samples to avoid the washing effect of analytes due to the high volume required in the case of seawater. The cartridges were first conditioned by percolating methanol (two cycles of 2.5 mL for 60 mg cartridges and 4 mL for 200 mg cartridges) under gravity conditions, followed by two more cycles of ultrapure water (2 mL and 4 mL for the 60 and 200 mg cartridges, respectively). After cartridge conditioning, the samples were loaded under vacuum conditions, and then the cartridges were dried under vacuum for 30 min. The extracts were eluted with two cycles of 2.5 or 4 mL of methanol for the extractions with the 60 or 200 mg cartridges. Finally, the extracts were evaporated to dryness under a gentle nitrogen stream at room temperature and reconstituted to 1.00 mL with methanol/water (10:90, *v*/*v*). The vials were kept at −20 °C until analysis.

### 3.6. Analysis of Antibiotics and Data Processing

The environmental samples were analysed by liquid chromatography coupled with high-resolution mass spectrometry (LC-HRMS), which is the technique of choice to investigate the occurrence of antibiotics and their TPs via wide-scope screening without reference standards.

The chromatographic separation was performed using an Aquity LC system (Waters^®^, Milford, MA, USA) equipped with a C18 analytical column (Hibar^®^ HR 50-21 Purospher^®^ STAR RP-18 end-capped column, Merck KGaA, Darmstadt, Germany) (3 μm, 2 × 125 mm), using mobile phases (A) acetonitrile and (B) HPLC water with 0.1% formic acid. The elution gradient started with 10% A, increasing to 99% in 8 min, holding to 10 min, and returning to initial conditions at the end of 12 min. Then, the conditions were held for one minute more. The flow rate was kept at 0.2 mL/min, and the initial injection volume was 10 µL. The chromatographic system was coupled to a Q-Exactive mass spectrometer (Thermo Fisher Scientific, San Jose, CA, USA) with an ESI source operating in positive mode. The data were acquired in full scan at 70,000 FWHM and a data-dependent scan (ddMS2) at 17,500 FWHM. During the analysis, the samples were kept at 15 °C. The acquisition parameters are summarised in Supplementary Material Table S8.

Data acquisition and processing were carried out using the Xcalibur Qual Browser software (Thermo Fisher Scientific, San Jose, CA, USA) and Compound Discoverer software version 3.3 SP1 from Thermo Fisher Scientific. The List S6 ITNANTIBIOTIC on NORMAN Suspect List Exchange [49], which includes data about the monoisotopic mass and formula of 677 antibiotics and their main transformation products (TPs), was included for the suspect screening. Furthermore, the information contained in two online databases, ChemSpider for structural information and MzCloud as a mass spectra database, was used. Supplementary Material Figure S1 summarises the workflow and tentative identification criteria.

### 3.7. Statistical Analysis and Visualisation

The clustered heatmap in Figure 2 was performed in R with R studio using pheatmap package version 1.0.12. The hierarchical cluster analysis was carried out using the squared Euclidean distance and the method of Ward was use for clustering.

In Figure 4a,b, the Pearson correlations were calculated and presented using the corrplot package in R version 1.2-9. https://CRAN.R-project.org/package=seriation (accessed on 15 November 2024).

In Figure 5, the data were previously normalised, the correlations were used according to the Pearson method, and missing data were not admitted. PCA analysis was carried out in R with R Studio Version 2023.12.1+402 and the libraries devtools and ggbiplot.

### 3.8. Quality Assurance and Quality Control

The chemical method used in the study was validated regarding linearity, sensitivity, accuracy, and repeatability of targeted compounds. Seven-point calibration curves were employed in the 0.5–100 µg/L range in vials, with a linearity range coefficient (R^2^) greater than 0.99 for the targeted antibiotics. Extraction recovery was calculated by dividing the peak area of the spiked samples by the peak area of the matrix-matched samples spiked in prepared wastewater. Wastewater was selected because it was the most complex matrix analysed in this study. The prepared wastewater that was used for recovery assessment comprised a mixture of real wastewater from different WWTPs. This mixture was extracted by SPE 2 times to avoid the initial levels of antibiotics. All of the compounds presented recoveries superior to 50%, and 82% presented recovery rates between 80–120%. The method repeatability was measured by intermediate precision, where the relative standard deviation (RSD, established by dividing the standard deviation value by the mean value of the measured concentration) at 10.0 and 50.0 ng/L was measured and was proven to be below 30% for all targets. The limit of detection (LOD) was in the range of 0.02–10 ng/L. To check for contamination of the whole analytical process, procedural blanks and field blanks were used, which underwent chemical analysis, together with the collected samples, and they were subtracted from the results.

## 4. Conclusions

Our study applies a non-targeted suspect screening analysis approach to assess major groups of antibiotics and ARGs in wastewater treatment plants and their receiving environments in two Mediterranean environments from NE and E of Spain (Ebro River and Ebro Delta, and Xúquer River and Albufera de València). Antibiotics were prioritised using a new approach to select those antibiotics that were most relevant to antimicrobial resistance spread. The results revealed that ARGs were found at similar levels during the different seasons, but a major number of ARGs was accounted for in summer samples, as was revealed through statistical correlations. Also, in the cases of *E. coli* and ESBL-*E. coli*, they presented similar levels across the different seasons in wastewater, but in surface water they were only detected in summer. On the other hand, major levels of antibiotics were found in winter, and the compounds that were detected at higher concentrations were metronidazole-OH, doxycycline, tilmicosin, ciprofloxacin, trimethoprim, tetracycline, norfloxacin, and ofloxacin.

## Figures and Tables

**Table 1:** Prioritisation criteria scores (0–4) for antimicrobials.

CRITERIA		0	1	2	3	4
**FREQUENCY (f) Of DETECTION**			<25	25–50	50–75	>75
**1/PNEC** *			<1	1 ≥ x < 10	10 ≥ x < 100	≥100
**∑** **(*I*)/8**	Individual pathogen contribution (*I*) **		<25%	25–50%	50–75%	>75%
**ANIMAL USE**	Antibiotics for animals in accordance with EMA categorisation	A (avoid)	B (restrict)	C (caution)	D (prudence)	-

* PNEC: predicted no effect concentration; ** *I*: individual isolates percentage.

**Table 2:** Range of concentrations above the limit of quantification (LOQ) for each type of sample.

Antimicrobials	WWTP Influent		WWTP Effluent		Surface Water		Seawater	
	Range (ng/L)	f (%)	Range (ng/L)	f (%)	Range (ng/L)	f (%)	Range (ng/L)	f (%)
Sulfamethazine	150–2041	55	184–1628	44	101–370	34	-	-
Sulfamethoxazole	20–10,622	76	26–17,906	60	26–407	22	333	14
Sulfapyridine	16–3935	61	9–1406	81	6–408	34	-	-
Trimethoprim	21–21,168	57	19–13,558	53	62–7949	34	-	-
Ciprofloxacin	682–22,071	97	160–7162	72	201–2057	34	-	-
Norfloxacin	489–18,944	76	2729–12,564	34	1207–15,692	34	-	-
Ofloxacin	278–10,992	97	132–7688	97	27–1055	41	-	-
Ampicillin	9–103	24	10–22	9	4–7	6	-	-
Amoxicillin	40–225	12	-	-	-	-	-	-
Clindamycin	18–671	24	11–622	50	12–157	31	-	-
Lincomycin	154–3679	36	13–733	31	33–639	34	-	-
Azithromycin	56–5597	55	43–6190	62	7–209	22	-	-
Clarithromycin	6–944	58	8–1120	66	0.3–57	12	-	-
Erythromycin	70–7070	42	35–2672	44	16–1534	28	-	-
Roxithromycin	25–32	9	10–18	6	-	-	-	-
Tilmicosin	302–78,998	33	46–1961	19	25–1058	22	-	-
Dimetridazole	44–1142	33	85–524	34	35–429	34	-	-
Metronidazole	200–9062	33	258–1237	34	16–230	31	-	-
Metronidazole OH	1289–104,945	36	338–4108	34	331–2538	34	-	-
Doxycycline	1641–92,223	12	28,948	3	12,344	3	-	-
Tetracycline	82–19,239	82	240–6828	69	23–2754	81	621–641	28

**f:** frequency of values > LOQ.

## Data Availability

https://www.norman-network.com/nds/bacteria/argSearchShow.php (accessed on 21 January 2025).

## Supplementary Materials

The following supporting information can be downloaded at: https://www.mdpi.com/article/10.3390/antibiotics14040341/s1, Figure S1: Identification levels applied in suspect screening of antibiotic residues in the samples. Table S1. Climatology conditions in the sampling areas. Table S2. List of samples and characteristics. Table S3. Levels of *E. coli* results and *E. coli* resistant to β-lactams results. Table S4. Summary of the detection frequency of antimicrobials. Table S5. Prioritisation scores calculated for each antimicrobial tentatively identified at confidence levels 2–3. Frequency of detection (f); minimum predicted no-effect concentration (PNEC); antibiotic resistance (AR) levels. Table S6. Relative recoveries (%) of target antibiotics in surface water (in brackets %RSD) spiked at 100 ng/L, the method limits of detection (MLOD) and quantification (MLOQ), monoisotopic mass, and main mass. Table S7. Target compounds and the isotopically labelled internal standards. Table S8. Acquisition parameters for the chemical analysis of antimicrobials. Table S9. Statistical significance of the different studied seasons. Complementary Table S1: Suspected screening per season. Complementary Table S2: Quantitative analysis of antimicrobials in the samples.
