# Supplementary material for: Comprehensive Study of Antibiotics and Antibiotic Resistance Genes in Wastewater and Impacted Mediterranean Water Environments
Source: Antibiotics (Basel). 2025 Mar 26;14(4):341. doi: 10.3390/antibiotics14040341 (PMC12024384; doi:10.3390/antibiotics14040341)
Supplement: Supplementary file 1 [file antibiotics-14-00341-s001.zip › antibiotics-3464791-supplementary.pdf]

COMPREHENSIVE STUDY OF ANTIBIOTICS AND ANTIBIOTIC RESISTANCE GENES IN WASTEWATER AND IMPACTED MEDITERRANEAN WATER ENVIRONMENTS

Maria Garcia-Torné[a,b] , Irene Falcó[c,d], Xavier Borrell[a], Arianna Bautista[a,b], Rachida Mazigh[a], Rosa Aznar[d], Gloria Sánchez[c], Marinella Farré[a] & Marta Llorca[a,*]

[a]Institute of Environmental Assessment and Water Research, C/ Jordi Girona, 18-26, 08034, Barcelona, Spain. mgtqam@cid.csic.es (M.G-T); xbdqam@cid.csic.es (X.B); abgqsh@cid.csic.es (A.B), rmlqsh@cid.csic.es (R.M.); mfuqam@cid.csic.es (M. F.); mlcqam@cid.csic.es (M.L.)

[b]Doctoral Program in Analytical Chemistry and Environmental Science, Dep. Of Chemical Engineering and Analytical Chemistry, University of Barcelona, 08028 Barcelona, Spain.

[c]VISAFELab Laboratory. Department of Preservation and Food Safety Technologies, IATA-CSIC, 46980 Valencia, Spain. irene.falco@iata.csic.es (I.F.); gloriasanchez@iata.csic.es (G.S.)

[d]Department of Microbiology and Ecology, University of Valencia, 46100 Valencia, Spain. Rosa.aznar@uv.es (R.A.)

***Corresponding authors:** marta.llorca@idaea.csic.es

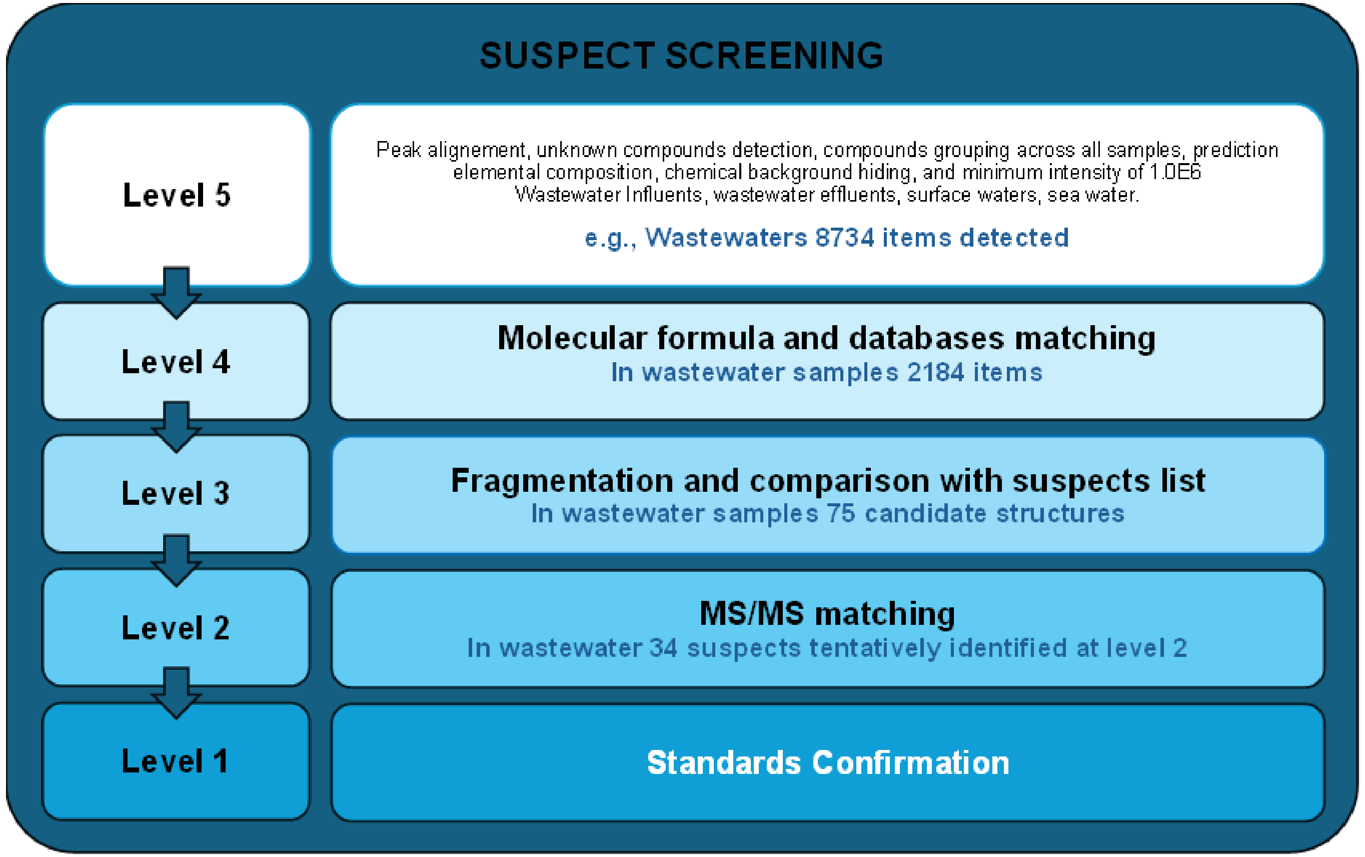

Fig. S1 | Identification levels applied in suspect screening of antibiotic residues in the samples.

Table S1. Levels of *E. coli* results and *E. coli* resistant to β-lactams results.

Code Influents	*E. coli* UFC/100mL	ESBL UFC/100mL	Code effluents	*E. coli* UFC/100mL	ESBL UFC/100mL
**WWTP-AS-I-W**	7.85	7.46	WWTP-AS-E-W	4.78	<LOD
**WWTP-Q-I-W**	7.43	6.48	WWTP-Q-E-W	4.48	4.00
**WWTP-P-I-W**	7.72	7.34	WWTP-P-E-W	<LOD	<LOD
**WWTP-ES-I-W**	7.23	6.00	WWTP-ES-E-W	<LOD	<LOD
**WWTP-SP-I-W**	7.43	6.95	WWTP-SP-E-W	5.11	4.00
**WWTP-AS-I-S**	6.45	5.60	WWTP-AS-I-S	<LOD	0.00
**WWTP-Q-I-S**	6.13	0.00	WWTP-Q-I-S	3.18	0.00
**WWTP-P-I-S**	7.38	6.15	WWTP-P-I-S	<LOD	0.00
**WWTP-ES-I-S**	6.43	5.74	WWTP-ES-I-S	<LOD	0.00
**WWTP-SP-I-S**	6.82	6.46	WWTP-SP-I-S	5.18	4.93
**WWTP-AS-I-Sp**	6.53	5.85	WWTP-AS-I-Sp	<LOD	<LOD
**WWTP-Q-I-Sp**	5.70	4.64	WWTP-Q-I-Sp	<LOD	<LOD
**WWTP-P-I-Sp**	6.33	3.18	WWTP-P-I-Sp	<LOD	<LOD
**WWTP-ES-I-Sp**	6.65	5.85	WWTP-ES-I-Sp	<LOD	<LOD
**WWTP-SP-I-Sp**	5.60	3.18	WWTP-AS-I-Sp	<LOD	<LOD

LOD: Limit of detection

Table S2| Summary of the detection frequency of antimicrobials.

Antimicrobial class	Antimicrobials	Wastewater samples						Surface water samples	
		Nº detections influents	Nº detections effluents	Total Nº detections	Frequency Influents	Frequency Effluents	Frequency wastewater	Detections in surface water	Frequency in surface water
**Ansamycins**	Rifaximin	3	0	3	**9.1**	**0.0**	**4.6**	-	**-**
**Aminocoumacin**	Amicoumacin A	4	0	4	**12.1**	**0.0**	**6.2**	-	**-**
**Aminoglycoside**	Netilmicin	3	0	3	**9.1**	**0.0**	**4.6**	-	**-**
	Etisomicin	5	4	9	**15.2**	**12.5**	**13.8**	5	**15.2**
	Istamycin A	3	2	5	**9.1**	**6.3**	**7.7**	-	**-**
	Spectinomycin	5	1	6	**15.2**	**3.1**	**9.2**	-	**-**
	Nebramycin 5'	6	4	10	**18.2**	**12.5**	**15.4**	3	**9.1**
	Sisomicin/Sisomycin	-	-	-	**-**	**-**	**-**	4	**12.1**
**Antifolates & sulfamides**	N4-Acetylsulfamethoxazole	1	0	1	**3.0**	**0.0**	**1.5**	-	**-**
	Sulfamethazine	24	15	39	**72.7**	**46.9**	**60.0**	14	**42.4**
	Sulfamethoxazole	30	6	36	**90.9**	**18.8**	**55.4**	9	**27.3**
	Trimethoprim	24	13	37	**72.7**	**40.6**	**56.9**	8	**24.2**
	Ormetoprim	1	0	1	**3.0**	**0.0**	**1.5**		
	Sulfaquinoxaline	1	0	1	**3.0**	**0.0**	**1.5**	2	**6.1**
	Sulfapyridine	21	14	35	**65.6**	**42.4**	**53.8**	6	**18.2**
**Beta-lactam**	Amoxicillin	15	14	29	**45.5**	**43.8**	**44.6**	-	**-**
	Ampicillin	15	11	26	**45.5**	**34.4**	**40.0**	-	**-**
	Epicillin	2	1	3	**6.1**	**3.1**	**4.6**	-	**-**
	Bacampicillin	1	0	1	**3.0**	**0.0**	**1.5**	-	**-**
**Carbapenem**	Panipenem	6	7	13	**18.2**	**21.9**	**20.0**	-	**-**
**Fluoroquinolone**	Ciprofloxacin	23	22	45	**69.7**	**68.8**	**69.2**	7	**21.2**
	Miloxacin	1	0	1	**3.0**	**0.0**	**1.5**	-	**-**
	Norfloxacin	26	7	33	**81.2**	**21.9**	**50.8**	7	**21.2**
	Ofloxacin	30	21	51	**90.9**	**65.6**	**78.5**	12	**36.4**
**Lincosamide**	Clindamycin	24	9	33	**75.0**	**27.2**	**50.8**	4	**12.1**
	Lincomycin	22	15	37	**66.7**	**46.9**	**56.9**	8	**24.2**

Table S2 (continuation)

Antimicrobial class	Antimicrobials	Wastewater samples						Surface water samples	
		Nº detections influents	Nº detections effluents	Total Nº detections	Frequency Influents	Frequency Effluents	Frequency wastewater	Detections in surface water	Frequency in surface water
Macrolide	Azithromycin	27	10	37	**81.8**	**31.3**	**56.9**	13	**39.4**
	Chalcomycin	5	0	5	**15.2**	**0.0**	**7.7**	-	**-**
	Clarithromycin	28	20	48	**84.8**	**62.5**	**73.8**	13	**39.4**
	Erythromycin	22	14	36	**66.7**	**43.8**	**55.4**	8	**24.2**
	Roxithromycin	18	19	37	**54.5**	**59.4**	**56.9**	4	**12.1**
	Spiramycin III	4	0	4	**12.1**	**0.0**	**6.2**	-	**-**
	Tilmicosin	29	14	43	**87.9**	**43.8**	**66.2**	9	**27.3**
Nitroimidazoles	Dimetridazole	33	12	45	**100.0**	**37.5**	**69.2**	5	**15.2**
	Methylimidazoleacetic acid	4	0	4	**12.1**	**0.0**	**6.2**	3	**9.1**
	Metronidazole	9	4	13	**27.3**	**12.5**	**20.0**	6	**18.2**
**Quinolone**	Ethoxyquin	-	-	-	**-**	**-**	**-**	3	**9.1**
**Tetracycline**	Tetracycline	28	17	45	**84.8**	**53.1**	**69.2**	19	**57.6**
	Alamycin	6	0	6	**18.2**	**0.0**	**9.2**	-	**-**
**Aminopeptidase**	Amastatin	2	2	4	**6.1**	**6.3**	**6.2**	-	**-**
	Bestatin/Ubenimex	6	5	11	**18.2**	**15.6**	**16.9**	-	**-**
**OTHER**	Aurantiogliocladin	3	2	5	**9.1**	**6.3**	**7.7**	-	**-**
	Mupirocin	1	0	1	**3.0**	**0.0**	**1.5**	-	**-**
	Resorcinomycin B	14	2	16	**42.4**	**6.3**	**24.6**	9	**27.3**
	Leptophyllin B	1	1	2	**3.0**	**3.1**	**3.1**	-	**-**
	Asteromycin	-	-	-	**-**	**-**	**-**	6	**18.2**
	Netropsin	-	-	-	**-**	**-**	**-**	6	**18.2**
	Tubercidin	-	-	-	**-**	**-**	**-**	6	**18.2**
	Imidocarb	-	-	-	**-**	**-**	**-**	6	**18.2**
	Diaveridine	-	-	-	**-**	**-**	**-**	1	**3.0**
**Other veterinary uses**	Pleuromutilin	-	-	-	**-**	**-**	**-**	6	**18.2**
	Salinomycin	-	-	-	**-**	**-**	**-**	6	**18.2**
**Antiviral agents**	Daclatasvir	5	4	9	**15.2**	12.5	13.8	-	**-**
	Moroxydine	1	0	1	**3.0**	0.0	1.5	-	**-**
	Raltegravir	2	1	3	**6.1**	3.1	4.6	-	**-**

Table S3 | Prioritisation scores calculated for each antimicrobial tentatively identified at levels 2-3 of confidence. Frequency of detection (f); mínimum predicted no-effect concentration (PNEC); antibiotic resistance levels (AR).

Gloup	Antimicrobial	f	*f - score*	PNEC	*PNEC - score*	*Acitenobacter baumannii*	*Enterococcus faecalis*	*Enterococcus faecium*	*Escherichia coli*	*Klebsiella pneumonidae*	*Pseudomonas aeriginosa*	*Streptococcus pneumoniae*	*Salmonella spp.*	*AR - score*	Use in veterinary medicine	TOTAL
**Ansamycins**	Rifaximin	4.6	0	0.0025	4				10					0	2	**6**
**Aminocoumacin**	Amicoumacin A	6.2	0	0.85	2									0	0	**2**
**Aminoglycoside**	Netilmicin	4.6	0	1.22	1	33	25	2.5		19.2	7.3			0.75	2	**3.75**
	Etisomicin	13.8	0	1.65	1	33	25	2.5		19.2	7.3			0.75	2	**3.75**
	Istamycin A	7.7	0	1.78	1	33	25	2.5		19.2	7.3			0.75	2	**3.75**
	Spectinomycin	9.2	0	22.2	0	33	25	2.5		19.2	7.3			0.75	2	**2.75**
	Nebramycin 5'	15.4	0	2.06	1	33	25	2.5		19.2	7.3			0.75	2	**3.75**
**Antifolates & sulfamides**	N4-Acetylsulfamethoxazole	1.5	0	2.36	1				40		80		25	0.875	3	**4.875**
	Sulfamethazine	60	4	0.6	2				40		80		25	0.875	3	**9.875**
	Sulfamethoxazole	55.4	4	0.03	3				40		80		25	0.875	3	**10.875**
	Trimethoprim	56.9	4	120	0	42			40	28	80		25	1.375	3	**8.375**
	Ormetoprim	1.5	0	2.51	1				40		80		25	0.875	3	**4.875**
	Sulfaquinoxaline	1.5	0	0.17	2				40		80		25	0.875	3	**5.875**
	Sulfapyridine	53.8	4	0.46	2				40		80		25	0.875	3	**9.875**
**Beta-lactams**	Amoxicillin	44.6	3	0.0037	4	20	0.6	87	13	44		21.6	23	1.375	3	**11.375**
	Ampicillin	40	3	0.012	3	20		87	71	91		21.6	23	1.75	3	**10.75**
	Epicillin	4.6	0	2.1	1	20	0.6	87	33	44		21.6	23	1.5	3	**5.5**
	Bacampicillin	1.5	0	0.3	2	20	0.6	87	33	44		21.6	23	1.5	3	**6.5**
**Carbapenems**	Panipenem	20	1	3.48	1	45	0.6	87	10	5.2	40	21.6	23	1.625	1	**4.625**
**Fluoroquinolones**	Ciprofloxacin	69.2	4	0.089	3	50		85	33	28.7	23.4		40	1.625	1	**9.625**
	Miloxacin	1.5	0	3.16	1	39.1		85	22	28.7	23.4		40	1.5	1	**3.5**
	Norfloxacin	50.8	4	0.16	2	39.1		85	22	28.7	23.4		40	1.5	1	**8.5**
	Ofloxacin	78.5	4	1.39	1	39.1		85	22	28.7	23.4		40	1.5	1	**7.5**
**Lincosamides**	Clindamycin	50.8	4	0.044	3	39.1	25	25	*					0.5	2	**9.5**
	Lincomycin	56.9	4	5.44	1	52	80	80	*					1.375	2	**8.375**

Gloup	Antimicrobial	f	*f - score*	PNEC	*PNEC - score*	*Acitenobacter baumannii*	*Enterococcus faecalis*	*Enterococcus faecium*	*Escherichia coli*	*Klebsiella pneumonidae*	*Pseudomonas aeriginosa*	*Streptococcus pneumoniae*	*Salmonella spp.*	*AR - score*	Use in veterinary medicine	TOTAL
**Macrolides**	Azithromycin	56.9	4	0.019	3		50	50	*			20.7	0.5/	0.75	2	**9.75**
	Chalcomycin	7.7	0	0.065	3		50	50	*			20.7	0.5/	0.75	2	**5.75**
	Clarithromycin	73.8	4	0.12	2		50	50	*			20.7	0.5/	0.75	2	**8.75**
	Erythromycin	55.4	4	0.3	2		50	87	*			20.7	0.5/	0.75	2	**8.75**
	Roxithromycin	56.9	4	0.0025	4		50	50	*			20.7	0.5/	0.75	2	**10.75**
	Spiramycin III	6.2	0	0.062	3		50	50	*			20.7	0.5/	0.75	2	**5.75**
	Tilmicosin	66.2	4	0.069	3		50	50	*			20.7	0.5/	0.75	2	**9.75**
**Nitroimidazoles**	Dimetridazole	69.2	4	1.43	1	50	70	70						1	3	**9**
	Methylimidazoleacetic acid	6.2	0	9.41	1									0	3	**4**
	Metronidazole	20	2	33.1	0	50	80	80				52	55	2	3	**7**
**Tetracyclines**	Tetracycline	69.2	4	0.06	3	5	20	10	10	17			23	1	3	**11**
	Alamycin	9.2	0	0.09	3		20	10	72.6				23	0.75	3	**6.75**
**Aminopeptidase**	Amastatin	6.2	0	1.1	1									0	0	**1**
	Bestatin/Ubenimex	16.9	0	26.5	0									0	0	**0**
**Other**	Aurantiogliocladin	7.7	0	26.5	0									0	0	**0**
	Mupirocin	1.5	0	1.87	1									0	0	**1**
	Resorcinomycin B	24.6	1	1.42	1									0	0	**2**
	Leptophyllin B	3.1	0	1.65	1									0	0	**1**

Table S4 | Relative recoveries (%) of target antibiotics in surfacewater (in brackets %RSD) spiked at 100 ng/l; method limits of detection (MLOD), and method limits of quantification (MLOQ); monoisotopic mass, and main mass.

Compound	Recovery (100 ng/l)	MLOD (ng/l)	MLOQ (ng/l)	Monoisotopic mass	Ion 1 (m/z)	Ion 2 (m/z)
**Amoxicillin**	79 (±3.2)	7.2	22.2	365.1045	349	114
**Ampicillin**	103 (±1.0)	1.1	3.5	349.1096	106	192
**Azithromycin**	96 (±15)	0.5	1.5	748.5085	591	116
**Ciprofloxacin**	98 (±9.8)	1.5	4.8	331.1332	288	245
**Clarithromycin**	90 (±1.5)	0.7	2.1	747.4769	158	596.4
**Clindamycin**	96 (±10)	1.0	3.1	424.1799	126.2	377
**Doxycycline**	85 (±1.8)	2.6	8.0	462.1638	154	429
**Erythromycin**	94 (±9.5)	0.7	2.2	733.4612	576	413
**Lincomycin**	105 (±5.9)	0.5	1.7	406.2138	359	389
**Metronidazole**	95 (±1.8)	0.8	2.5	171.0644	128	82
**Metronidazole-OH**	80(±2.7)	0.4	1.2	187.0593	126	123
**Norfloxacin**	96 (±15)	4.0	12	319.1332	276.2	233
**Ofloxacin**	99 (±15)	1.5	4.6	361.1438	318	261
**Roxythromycin**	87(±5.7)	0.8	2.6	836.5246	679	158
**Sulfamethazine**	92 (±1.1)	0.6	1.9	278.0837	186	91.9
**Sulfamethoxazole**	95 (±1.0)	0.4	1.2	253.0521	156	92
**Sulfapyridine**	75 (±25)	1.5	4.5	249.0572	156	92
**Tetracycline**	110 (±11)	2.0	6.0	444.1533	410	154
**Tilmicosin**	90 (±15)	3.6	11	868.566	245	189
**Trimethoprim**	105 (±2.6)	1.5	5.0	290.1379	230	261

Table S5. Climatology conditions in the sampling areas,

Sampling campaigns		Average temperatures (ºC)	Average precipitation (mm)	Average solar irradiation (kWh/m^2)**	References
Winter (January 2022)	Zaragoza	-2-15	< 50	2.62	[59]
	Ebro Delta	0-20	200-300	2.69	[57][62]
	Albufera	6-19	≤ 50	2.75	[58]*
Summer (July 2022)	Zaragoza	15-37	< 50	7.66	[59]
	Ebro Delta	15-35	< 50	7.35	[57][62]
	Albufera	19-32	< 20	7.40	[58]*
Rainy Season (March 2023)	Zaragoza	8-18	<50	4.80	[59]
	Ebro Delta	11-16	<50	4.58	[60][61]
	Albufera	9-19	≤ 50	5.12	[58]*

*Referred to Silla, Valencia.

** From AEMET (https://www.aemet.es/en/portada),

Table S6 | List of samples and characteristics.

Type of sample	Sampling period	Sampling site	Code	Coordinates	T	pH	COD	WWTP eq. population	WWTP treatments	Characteristics
**Wastewater influent**	17/18-jan-22	La Cartuja (Zaragoza)	WWTP-LC-I-W	41.6679, -0.9261	11	-	-	1,200.000	1-3	Biological P elimination.
**Wastewater influent**	17/18-jan-22	La Almazara (Zaragoza)	WWTP-LA-I-W	41.6099, -0.8110	11	-	-	100.000	1-2	Biological P elimination.
**Wastewater influent**	17/18-jan-22	Flix	WWTP-F-I-W	41.2391, 0.5519	7	-	-	12.928	1-2	Biological P elimination.
**Wastewater influent**	17/18-jan-22	Tortosa-Roquetas	WWTP-TR-I-W	40.7961, 0.5087	9	-	-	46.847	1-2	Biological N/P elimination.
**Wastewater influent**	17/18-jan-22	Amposta	WWTP-A-I-W	40.7038, 0.6101	10	-	-	27.500	1-2	Biological
**Wastewater influent**	17/18-jan-22	St Carles de la Ràpita	WWTP-StC-I-W	40.6271, 0.6220	10	-	-	28.921	1-3	Biological N elimination.
**Wastewater influent**	24/25-jan-22	Albufera Sur	WWTP-AS-I-W	39.2708, -0.4029	11	-	-	198.916	1-2	N and P elimination.
**Wastewater influent**	24/25-jan-22	Quart	WWTP-Q-I-W	39.4546, -0.4134	11	-	-	412.500	1-3	N and P elimination. UV.
**Wastewater influent**	24/25-jan-22	Pinedo	WWTP-P-I-W	39.4423, -0.3442	11	-	-	942.240	1-3	UV
**Wastewater influent**	24/25-jan-22	El Saler	WWTP-ES-I-W	39.3629, -0.3281	11	-	-	14.000	1-2	N and P elimination. UV.
**Wastewater influent**	24/25-jan-22	Sueca-Perelló	WWTP-SP-I-W	39.2742, -0.2810	11	-	-	45.783	1-3	N and P elimination. UV.
**Wastewater effluent**	17/18-jan-22	La Cartuja (Zaragoza)	WWTP-LC-E-W	41.6679, -0.9261	11	-	-	1,200.000	1-3	Biological P elimination.
**Wastewater effluent**	17/18-jan-22	La Almazara (Zaragoza)	WWTP-LA-E-W	41.6099, -0.8110	11	-	-	100.000	1-2	Biological P elimination.
**Wastewater effluent**	17/18-jan-22	Flix	WWTP-F-E-W	41.2391, 0.5519	7	-	-	12.928	1-2	Biological P elimination.
**Wastewater effluent**	17/18-jan-22	Tortosa-Roquetas	WWTP-TR-E-W	40.7961, 0.5087	9	-	-	46.847	1-2	Biological N/P elimination.
**Wastewater effluent**	17/18-jan-22	Amposta	WWTP-A-E-W	40.7038, 0.6101	10	-	-	27.500	1-2	Biological
**Wastewater effluent**	17/18-jan-22	St Carles de la Ràpita	WWTP-StC-E-W	40.6271, 0.6220	10	-	-	28.921	1-3	Biological N elimination.
**Wastewater effluent**	24/25-jan-22	Albufera Sur	WWTP-AS-E-W	39.2708, -0.4029	11	-	-	198.916	1-2	N and P elimination.
**Wastewater effluent**	24/25-jan-22	Quart	WWTP-Q-E-W	39.4546, -0.4134	11	-	-	412.500	1-3	N and P elimination. UV.
**Wastewater effluent**	24/25-jan-22	Pinedo	WWTP-P-E-W	39.4423, -0.3442	11	-	-	942.240	1-3	UV
**Wastewater effluent**	24/25-jan-22	El Saler	WWTP-ES-E-W	39.3629, -0.3281	11	-	-	14.000	1-2	N and P elimination. UV.
**Wastewater effluent**	24/25-jan-22	Sueca-Perelló	WWTP-SP-E-W	39.2742, -0.2810	11	-	-	45.783	1-3	N and P elimination. UV.
**Ebro River**	17/18-jan-22	La Cartuja	ER-LC-W	41.5813, -0.7600	7.1	6.24	28.80	-	-	-

Type of sample	Sampling period	Sampling site	Code	Coordinates	T	pH	COD	WWTP eq. population	WWTP treatments	Characteristics
**Ebro River**	17/18-jan-22	La Almazara	ER-LA-W	41.6505, -0.8552	7.4	6.68	14.60	-	-	-
**Ebro River**	17/18-jan-22	Flix	ER-F-W	41.2453, 0.5561	7.3	5.84	19.71	-	-	-
**Ebro River**	17/18-jan-22	Tortosa	ER-T-W	40.7748, 0.5348	9.7	7.26	12.85	-	-	-
**Ebro River**	17/18-jan-22	Amposta	ER-A-W	40.7042, 0.6192	9.6	6.50	16.92	-	-	-
**Albufera**	26/27-jan-22	Albufera P1	ALB-P1-W	39.4221,-0.3530	13.6	6.76	11.64	-	-	-
**Albufera**	26/27-jan-22	Albufera P2	ALB-P2-W	39.3060,-0.3588	11.3	6.67	13.68	-	-	-
**Albufera**	26/27-jan-22	Albufera P3	ALB-P3-W	39.3939,-0.3506	9.7	6.83	11.39	-	-	-
**Albufera**	26/27-jan-22	Albufera P4	ALB-P4-W	39.3814,-0.3397	9.3	6.77	14.79	-	-	-
**Albufera**	26/27-jan-22	Albufera P5	ALB-P5-W	39.3495,-0.3235	10.6	6.69	14.26	-	-	-
**Albufera**	26/27-jan-22	Albufera P6	ALB-P6-W	39.3275,-0.3184	9.8	7.20	14.49	-		-
**Wastewater influent**	12-jul-22	La Cartuja (Zaragoza)	WWTP-LC-I-S	41.6679, -0.9261	-	-	-	1,200.000	1-3	Biological P elimination.
**Wastewater influent**	13-jul-22	La Almazara (Zaragoza)	WWTP-LA-I-S	41.6099, -0.8110	-	-	-	100.000	1-2	Biological P elimination.
**Wastewater influent**	13-jul-22	Flix	WWTP-F-I-S	41.2391, 0.5519	-	-	-	12.928	1-2	Biological P elimination.
**Wastewater influent**	13-jul-22	Tortosa-Roquetas	WWTP-TR-I-S	40.7961, 0.5087	-	-	-	46.847	1-2	Biological N/P elimination.
**Wastewater influent**	13-jul-22	Amposta	WWTP-A-I-S	40.7038, 0.6101	-	-	-	27.500	1-2	Biological
**Wastewater influent**	13-jul-22	St Carles de la Ràpita	WWTP-StC-I-S	40.6271, 0.6220	-	-	-	28.921	1-3	Biological N elimination.
**Wastewater influent**	04/05-jul-22	Albufera Sur	WWTP-AS-I-S	39.2708, -0.4029	-	-	-	198.916	1-2	N and P elimination.
**Wastewater influent**	04/05-jul-22	Quart	WWTP-Q-I-S	39.4546, -0.4134	-	-	-	412.500	1-3	N and P elimination. UV.
**Wastewater influent**	04/05-jul-22	Pinedo	WWTP-P-I-S	39.4423, -0.3442	-	-	-	942.240	1-3	UV
**Wastewater influent**	04/05-jul-22	El Saler	WWTP-ES-I-S	39.3629, -0.3281	-	-	-	14.000	1-2	N and P elimination. UV.
**Wastewater influent**	04/05-jul-22	Sueca-Perelló	WWTP-SP-I-S	39.2742, -0.2810	-	-	-	45.783	1-3	N and P elimination. UV.
**Wastewater effluent**	04/05-jul-22	La Cartuja (Zaragoza)	WWTP-LC-E-S	41.6679, -0.9261	-	-	-	1,200.000	1-3	Biological P elimination.
**Wastewater effluent**	04/05-jul-22	La Almazara (Zaragoza)	WWTP-LA-E-S	41.6099, -0.8110	-	-	-	100.000	1-2	Biological P elimination.
**Wastewater effluent**	04/05-jul-22	Flix	WWTP-F-E-S	41.2391, 0.5519	-	-	-	12.928	1-2	Biological P elimination.
**Wastewater effluent**	04/05-jul-22	Tortosa-Roquetas	WWTP-TR-E-S	40.7961, 0.5087	-	-	-	46.847	1-2	Biological N/P elimination.
**Wastewater effluent**	04/05-jul-22	Amposta	WWTP-A-E-S	40.7038, 0.6101	-	-	-	27.500	1-2	Biological

Type of sample	Sampling period	Sampling site	Code	Coordinates	T	pH	COD	WWTP eq. population	WWTP treatments	Characteristics
**Wastewater effluent**	04/05-jul-22	St Carles de la Ràpita	WWTP-StC-E-S	40.6271, 0.6220	-	-	-	28.921	1-3	Biological N elimination.
**Wastewater effluent**	04/05-jul-22	Albufera Sur	WWTP-AS-E-S	39.2708, -0.4029	-	-	-	198.916	1-2	N and P elimination.
**Wastewater effluent**	04/05-jul-22	Quart	WWTP-Q-E-S	39.4546, -0.4134	-	-	-	412.500	1-3	N and P elimination. UV.
**Wastewater effluent**	04/05-jul-22	Pinedo	WWTP-P-E-S	39.4423, -0.3442	-	-	-	942.240	1-3	UV
**Wastewater effluent**	04/05-jul-22	El Saler	WWTP-ES-E-S	39.3629, -0.3281	-	-	-	14.000	1-2	N and P elimination. UV.
**Wastewater effluent**	04/05-jul-22	Sueca-Perelló	WWTP-SP-E-S	39.2742, -0.2810	-	-	-	45.783	1-3	N and P elimination. UV.
**Ebro River**	12-jul-22	La Cartuja	ER-LC-S	41.5813, -0.7600	27.0	7.62	6.66	-	-	-
**Ebro River**	12-jul-22	La Almazara	ER-LA-S	41.6505, -0.8552	25.1	8.18	7.59	-	-	-
**Ebro River**	13-jul-22	Flix	ER-F-S	41.2453, 0.5561	24.7	7.81	7.42	-	-	-
**Ebro River**	13-jul-22	Tortosa	ER-T-S	40.7748, 0.5348	26.6	7.68	7.60	-	-	-
**Ebro River**	13-jul-22	Amposta	ER-A-S	40.7042, 0.6192	28.3	7.76	10.15	-	-	-
**Albufera**	6-jul-22	Albufera P1	ALB-P1-S	39.4221, -0.3530	25.6	8.33	17.09	-	-	-
**Albufera**	6-jul-22	Albufera P2	ALB-P2-S	39.3060, -0.3588	28.2	7.87	6.69	-	-	-
**Albufera**	6-jul-22	Albufera P3	ALB-P3-S	39.3939, -0.3506	31.4	7.84	13.47	-	-	-
**Albufera**	6-jul-22	Albufera P4	ALB-P4-S	39.3814, -0.3397	28.2	7.87	6.69	-	-	-
**Albufera**	6-jul-22	Albufera P5	ALB-P5-S	39.3495, -0.3235	28.5	7.98	8.45	-	-	-
**Albufera**	6-jul-22	Albufera P6	ALB-P6-S	39.3275, -0.3184	29.2	7.87	8.52	-	-	-
**Seawater**	13-jul-22	Els Alfacs 1	EA-P1-S	40.6181, 0.6120	30.0	6.30	-	-	-	-
**Seawater**	13-jul-22	Els Alfacs 2	EA-P2-S	40.6137, 0.6231	29.9	6.60	-	-	-	-
**Seawater**	13-jul-22	Els Alfacs 3	EA-P3-S	40.6071, 0.6149	29.6	6.60	-	-	-	-
**Seawater**	13-jul-22	Els Alfacs 4	EA-P4-S	40.5910, 0.5992	29.3	6.90	-	-	-	-
**Seawater**	13-jul-22	El Fangar 1	EF-P1-S	40.7983, 0.7082	29.0	…	-	-	-	-
**Seawater**	13-jul-22	El Fangar 2	EF-P2-S	40.7810, 0.7316	30.1	6.90	-	-	-	-
**Seawater**	13-jul-22	El Fangar 3	EF-P3-S	40.8082, 0.7352	29.4	7.20	-	-	-	-
**Seawater**	13-jul-22	El Fangar 4	EF-P4-S	40.8139, 0.7599	28.5	7.00	-	-	-	-
**Wastewater influent**	21-mar-23	La Cartuja (Zaragoza)	WWTP-LC-I-Sp	41.6679, -0.9261	-	-	-	1,200.000	1-3	Biological P elimination.
**Wastewater influent**	21-mar-23	La Almazara (Zaragoza)	WWTP-LA-I-Sp	41.6099, -0.8110	-	-	-	100.000	1-2	Biological P elimination.
**Wastewater influent**	21-mar-23	Flix	WWTP-F-I-Sp	41.2391, 0.5519	-	-	-	12.928	1-2	Biological P elimination.

Type of sample	Sampling period	Sampling site	Code	Coordinates	T	pH	COD	WWTP eq. population	WWTP treatments	Characteristics
**Wastewater influent**	22-mar-23	Tortosa-Roquetas	WWTP-TR-I-Sp	40.7961, 0.5087	-	-	-	46.847	1-2	Biological N/P elimination.
**Wastewater influent**	22-mar-23	Amposta	WWTP-A-I-Sp	40.7038, 0.6101	-	-	-	27.500	1-2	Biological
**Wastewater influent**	23-mar-23	St Carles de la Ràpita	WWTP-StC-I-Sp	40.6271, 0.6220	-	-	-	28.921	1-3	Biological N elimination.
**Wastewater influent**	23-mar-23	Albufera Sur	WWTP-AS-I-Sp	39.2708, -0.4029	-	-	-	198.916	1-2	N and P elimination.
**Wastewater influent**	23-mar-23	Quart	WWTP-Q-I-Sp	39.4546, -0.4134	-	-	-	412.500	1-3	N and P elimination. UV.
**Wastewater influent**	23-mar-23	Pinedo	WWTP-P-I-Sp	39.4423, -0.3442	-	-	-	942.240	1-3	UV
**Wastewater influent**	23-mar-23	El Saler	WWTP-ES-I-Sp	39.3629, -0.3281	-	-	-	14.000	1-2	N and P elimination. UV.
**Wastewater influent**	23-mar-23	Sueca-Perelló	WWTP-SP-I-Sp	39.2742, -0.2810	-	-	-	45.783	1-3	N and P elimination. UV.
**Wastewater effluent**	21-mar-23	La Cartuja (Zaragoza)	WWTP-LC-E-Sp	41.6679, -0.9261	-	-	-	1,200.000	1-3	Biological P elimination.
**Wastewater effluent**	21-mar-23	La Almazara (Zaragoza)	WWTP-LA-E-Sp	41.6099, -0.8110	-	-	-	100.000	1-2	Biological P elimination.
**Wastewater effluent**	21-mar-23	Flix	WWTP-F-E-Sp	41.2391, 0.5519	-	-	-	12.928	1-2	Biological P elimination.
**Wastewater effluent**	22-mar-23	Tortosa-Roquetas	WWTP-TR-E-Sp	40.7961, 0.5087	-	-	-	46.847	1-2	Biological N/P elimination.
**Wastewater effluent**	22-mar-23	Amposta	WWTP-A-E-Sp	40.7038, 0.6101	-	-	-	27.500	1-2	Biological
**Wastewater effluent**	23-mar-23	St Carles de la Ràpita	WWTP-StC-E-Sp	40.6271, 0.6220	-	-	-	28.921	1-3	Biological N elimination.
**Wastewater effluent**	23-mar-23	Albufera Sur	WWTP-AS-E-Sp	39.2708, -0.4029	-	-	-	198.916	1-2	N and P elimination.
**Wastewater effluent**	23-mar-23	Quart	WWTP-Q-E-Sp	39.4546, -0.4134	-	-	-	412.500	1-3	N and P elimination. UV.
**Wastewater effluent**	23-mar-23	Pinedo	WWTP-P-E-Sp	39.4423, -0.3442	-	-	-	942.240	1-3	UV
**Wastewater effluent**	23-mar-23	El Saler	WWTP-ES-E-Sp	39.3629, -0.3281	-	-	-	14.000	1-2	N and P elimination. UV.
**Wastewater effluent**	23-mar-23	Sueca-Perelló	WWTP-SP-E-Sp	39.2742, -0.2810	-	-	-	45.783	1-3	N and P elimination. UV.
**Ebro River**	21-mar-23	La Cartuja	ER-LC-Sp	41.5813, -0.7600	-	-	-	-	-	-
**Ebro River**	21-mar-23	La Almazara	ER-LA-Sp	41.6505, -0.8552	-	-	-	-	-	-
**Ebro River**	21-mar-23	Flix	ER-F-Sp	41.2453, 0.5561	-	-	-	-	-	-
**Ebro River**	22-mar-23	Tortosa	ER-T-Sp	40.7748, 0.5348	-	-	-	-	-	-
**Ebro River**	22-mar-23	Amposta	ER-A-Sp	40.7042, 0.6192	-	-	-	-	-	-

Type of sample	Sampling period	Sampling site	Code	Coordinates	T	pH	COD	WWTP eq. population	WWTP treatments	Characteristics
**Albufera**	23-mar-23	Albufera P1	ALB-P1-Sp	39.4221, -0.3530	-	-	-	-	-	-
**Albufera**	23-mar-23	Albufera P2	ALB-P2-Sp	39.3060, -0.3588	-	-	-	-	-	-
**Albufera**	23-mar-23	Albufera P3	ALB-P3-Sp	39.3939, -0.3506	-	-	-	-	-	-
**Albufera**	23-mar-23	Albufera P4	ALB-P4-Sp	39.3814, -0.3397	-	-	-	-	-	-
**Albufera**	23-mar-23	Albufera P5	ALB-P5-Sp	39.3495, -0.3235	-	-	-	-	-	-
**Albufera**	23-mar-23	Albufera P6	ALB-P6-Sp	39.3275, -0.3184	-	-	-	-	-	-

Table S7 | Target compounds and the isotopically labeled internal standards.

Compound	Compound formula	Molecular weight*	Standard preparation solvent	Corresponding internal standard	Internal standard formula	Molecular weight
Erythromycin	$C_{37}H_{67}NO_{13}$	733.93	Methanol	Erythromycin-d3	$C_{37}H_{64}D_3NO_{13}$	736.95
Azithromycin	$C_{38}H_{72}N_2O_{12}$	748.98	DMSO	Azithromycin-d3	$C_{38}H_{69}D_3N_2O_{12}$	752
Clarithromycin	$C_{38}H_{69}NO_{13}$	747.95	Methanol	Clarithromycin-N-methyl-d3	$C_{38}H_{66}D_3NO_{13}$	750.97
Tetracycline	$C_{22}H_{24}N_2O_8$	444.44	Methanol	Tetracycline-d6 (>80%)	$C_{22}H_{18}D_6N_2O_8$	450.47
Ofloxacin	$C_{18}H_{20}FN_3O_4$	361.37	Methanol + NaOH	Ofloxacin-d8	$C_{18}H_{12}D_8FN_3O_4$	369.42
Ciprofloxacin	$C_{17}H_{18}FN_3O_3$	331.34	Methanol + NaOH	Ciprofloxacin-d8	$C_{17}H_{10}D_8FN_3O_3$	339.39
Sulfamethoxazole	$C_{10}H_{11}N_3O_3S$	253.28	DMSO	Sulfamethoxazole-d4	$C_7H_7D_4N_3O_3S$	257.3
Trimethoprim	$C_{14}H_{18}N_4O_3$	290.32	Methanol	Trimethoprim-d3	$C_{14}H_{15}D_3N_4O_3$	293.34
Metronidazole OH[a]	$C_6H_9N_3O_4$	187.15	DMSO	Hydroxy Metronidazole-d4	$C_6H_5D_4N_3O_4$	191.18
Dimetridazole	$C_5H_7N_3O_2$	141.13	Methanol	Dimetridazol-d3	$C_5H_4D_3N_3O_2$	144.15
Norfloxacin	$C_{16}H_{18}FN_3O_3$	319.33	Methanol	Norfloxacin-d8	$C_{16}H_{10}D_8FN_3O_3$	327.38
Tilmicosin	$C_{46}H_{80}N_2O_{13}$	869.13	Methanol	Tilmicosin-d3	$C_{46}H_{77}D_3N_2O_{13}$	872.15
Sulfamethazine	$C_{12}H_{14}N_4O_2S$	278.33	DMSO	Sulfamethazine-d4	$C_{12}H_{10}D_4N_4O_2S$	282.35
Sulfapyridine	$C_{11}H_{11}N_3O_2S$	249.29	DMSO	Sulfapyridine-d4	$C_{11}H_7D_4N_3O_2S$	253.31
Lincomycin	$C_{18}H_{34}N_2O_6S$	406.54	Methanol	Lincomycin-d3	$C_{18}H_{31}D_3N_2O_6S$	409.56
Clindamycin	$C_{18}H_{33}ClN_2O_5S$	424.98	Methanol	Clindamycin-13C. D3	$C_{17}{}^{13}CH_{30}D_3ClN_2O_5S$	428.99
Metronidazole	$C_6H_9N_3O_3$	171.15	DMSO	Metronidazole-d4	C6H5D4N3O3	175.18
Doxycycline Hyclate	$C_{23}H_{29}ClN_2O_9$	512.94	Methanol	Doxycycline-d3 Hyclate (Major)	$2(C_{22}H_{21}D_3N_2O_8) \bullet 2HCl \bullet xH_2O \bullet C_2H_6O$	2(447.45) + 2(36.46) + x(18.02) + (46.07)
Roxithromycin	$C_{41}H_{76}N_2O_{15}$	837.05	DMSO	Roxithromycin-d7	$C_{41}H_{69}D_7N_2O_{15}$	844.09

*Refers to the unlabelled species.

Solvents such as water (HPLC grade) and methanol were from LiChrosolv (Darmstadt, Germany). NaOH (≥98% purity), Na_2EDTA (99% purity), and DMSO were provided by Sigma Aldrich (Steinheim, Germany). HCl was supplied by PanReac AppliChem (Murcia, Spain). ITW Reagents (Castellar del Vallès, Spain).

Glass microfibre filters of 0.70 µm pore size were acquired from Whatman (GF/F. Whatman™, Maidstone, UK).

Table S8 | Acquisition parameters for the chemical analysis of antimicrobials.

**Column**	C18 analytical column (Hibar® HR 50-21 Purospher® STAR RP-18 end-capped column) (3 µm, 2×125 mm)
**Sample temperature**	15 ºC
**Mobile phase A**	Acetonitrile
**Mobile phase B**	Water + formic acid 0.1%
**Gradient**	A from 50 % to 99 %
**Run time**	13 min
**Flow**	0.2 ml/min
**Injection volume**	10 µl
**Spectometer**	Q Exactive orbitrap
**Ionisation source**	ESI +
**Ionisation voltage**	3500.0 V
**Capillar temperature**	350 ºC
**Vaporizer temperature**	300 ºC
**Sweep gas (Aritrary units)**	10
**Sheath gas (Arbitrary units)**	10
**Collision voltage (arbitrary units)**	30
**Adquisition mode**	Full scan and data dependant scan ($ddMS^2$)
**Mass range**	100.0-1000.0 m/z
**Full MS Resolution**	70,000
**$ddMS^2$ Resolution**	17,500 FWHM

Table S9: Statistical significance of the different studied seasons

	F-statistic	p-value
**Sul1_1**	10.645953025679384	6.935717814620771e-05
**Sul2_1**	13.299081647134654	8.446051951059743e-06
**pbp2b**	8.136933312689	0.0005573812107166307
**blaCTX-M**	11.62244578758527	3.159676426072079e-05
**cmlA_2**	23.41313350158454	6.033153187160932e-09
**ermB_1**	13.895238415049679	5.3310720289561285e-06
**ermA**	9.39454004847195	0.00019381173330423922
**nimE**	9.25247906167906	0.00021811166781247467
**tetPB_3**	7.131651954790364	0.001320102274840389
**tetA_1**	7.9219222805470055	0.0006693502288571722
**emrB/qacA_1Sul1_1**	5.252758704065947	0.006916190070439031

References

Cuevas-Ferrando, E., Randazzo, W., Perez-Cataluna, A. and Sanchez, G. (2019) HEV Occurrence in Waste and Drinking Water Treatment Plants. Front Microbiol 10, 2937.

Gros, M., Rodriguez-Mozaz, S. and Barcelo, D. (2012) Fast and comprehensive multi-residue analysis of a broad range of human and veterinary pharmaceuticals and some of their metabolites in surface and treated waters by ultra-high-performance liquid chromatography coupled to quadrupole-linear ion trap tandem mass spectrometry. J Chromatogr A 1248, 104-121.

Perez-Cataluna, A., Cuevas-Ferrando, E., Randazzo, W., Falco, I., Allende, A. and Sanchez, G. (2021) Comparing analytical methods to detect SARS-CoV-2 in wastewater. Sci Total Environ 758, 143870.
